# International Conference on Genome Informatics (GIW 2013) in Singapore: introduction to the systems biology contributions

**DOI:** 10.1186/1752-0509-7-S6-I1

**Published:** 2013-12-13

**Authors:** Frank Eisenhaber, Wing-Kin Sung, Limsoon Wong

**Affiliations:** 1 Bioinformatics Institute, Agency for Science, Technology and Research, 30 Biopolis Street #07-01, Matrix, Singapore 138671; 2 Department of Biological Sciences, National University of Singapore, 8 Medical Drive, Singapore 117597; 3 School of Computer Engineering, Nanyang Technological University, 50 Nanyang Drive, Singapore 637553; 4 School of Computing, National University of Singapore, 13 Computing Drive, Singapore 117417; 5 Genome Institute of Singapore, 60 Biopolis Street #02-01, Genome, Singapore 138672

## 

The "International Conference on Genome Informatics", popularly known as "GIW", is probably one of the oldest, if not actually the oldest annual, regular conference in computational biology that survived all turns of the tempestuous development of this field of research [1]. It is impossible to overestimate its role for establishing and enhancing the computational biology and bioinformatics research community in the Asia-Pacific region and its interaction with the world-wide research effort. Importantly, it has provided a friendly forum where scientists especially from the region could exchange and publish their research findings. It has accompanied and furthered the growth of computational biology and bioinformatics research in both quantity and quality in the Asia-Pacific region.

The GIW was first held as an open workshop ("**G**enome **I**nformatics **W**orkshop", thus, GIW) at Kikai Shinko Kaikan in Tokyo during December 3-4, 1990, essentially just before the Japanese Human Genome Project started in the next year. Whereas GIW was originally an intra-Japanese affair, it changed to an international conference in 1993 and the currently used name of the conference was adopted in 2001. During the last ~15 years, the conference was always attended by several hundred participants; thus, it is not really a "workshop" any longer. Whereas GIW had more the role of a regional incubator in the early years, it has recently become one of the important, truly international conference venues in the bioinformatics field for scientific exchange. It provides unique opportunities to bridge theory and experiment, academia and industry, science from the East and the West.

The conference site was in Tokyo or Yokohama exclusively until 2006 (as well as 2009). GIW 2007 (the 18^th ^edition) was the first one held outside Japan, in the Biopolis in Singapore. Other locations in the Asia-Pacific regions were to follow: the 19^th ^GIW at the Gold Coast in Australia (2008), the 21^st ^GIW in Hangzhou (China) in 2010, the 22^nd ^GIW in Busan (South Korea) in 2011 and the 23^rd ^GIW in Tainan (Taiwan).

Remarkably, the 24^th ^GIW has been awarded to Singapore again [2] and, notably, is held in the same premises as the conference in 2007, namely in the Matrix Building of Biopolis. Singaporean bioinformaticians might tend to see this as recognition for their research efforts during the last years; though, the geographically central location in the Asia-Pacific region, the excellent transport hub and the infrastructural support of Singapore will lend an alternative, equally important explanation. All events happen only if an activist champions them. The Singaporean community is grateful to Limsoon Wong for his lobbying effort to attract important conferences here.

Given the maturity of the research area and today's scientific fashions, efforts that classify as system biology occupy a prominent place in GIW 2013. In total, eighteen submissions have qualified for this special issue of BMC Systems Biology. The systems biology approach aims at a holistic perspective, to explain and to predict phenotypic properties that are influenced by a multitude of factors with complex theoretical, desirably quantitative models.

Given the absence of a consistent, predictive biological theory as physicists have been used to since many decades, some might consider the quest for an integrated, system approach grandiloquent and premature. There are serious arguments for this view such as that about 50% of all eukaryote genes lack even tentative functional characterizations and, most likely, not even half of the biomolecular mechanisms are known [3]. Despite full genome sequencing, even a stable reference proteome cannot be deduced [4]. Thus, quantitative and predictive biology has a long way to go.

Nevertheless, the large-scale experimental techniques, most prominently nucleic acid sequencing but also epigenetics analyses, large-scale expression studies, proteomics with the large sets of protein-protein interaction data, the ever growing library of biomacromolecular structures and automated methods for analyzing cellular and tissue images [5] open new opportunities and, for carefully selected questions, interesting and important insights can be deduced from this data at the systems level that can even reach out into biomedical and biotechnological applications. The papers collected in this special edition exemplify how far research has moved forward.

## Competing interests

The authors declare that they have no competing interests.
